# Genomic Comparison among Lethal Invasive Strains of *Streptococcus pyogenes* Serotype M1

**DOI:** 10.3389/fmicb.2017.01993

**Published:** 2017-10-23

**Authors:** Gabriel R. Fernandes, Aulus E. A. D. Barbosa, Renan N. Almeida, Fabíola F. dos S. Castro, Marina de C. P. da Ponte, Celio Faria-Junior, Fernanda M. P. Müller, Antônio A. B. Viana, Dario Grattapaglia, Octavio L. Franco, Sérgio A. Alencar, Simoni C. Dias

**Affiliations:** ^1^Programa de Pós-Graduação em Ciências Genômicas e Biotecnologia, Universidade Católica de Brasília, Brasília, Brazil; ^2^Hospital Santa Luzia, Brasília, Brazil; ^3^Centro Universitário de Brasília–UniCEUB, Brasília, Brazil; ^4^Laboratório Central de Saúde Pública LACEN, Brasília, Brazil; ^5^Empresa Brasileira de Pesquisa Agropecuária, Embrapa Recursos Genéticos e Biotecnologia, Brasília, Brazil

**Keywords:** *Streptococcus pyogenes*, streptococcal toxic shock syndrome, invasive infection outbreak, *emm*, virulence factor, prophage

## Abstract

*Streptococcus pyogenes*, also known as group A *Streptococcus* (GAS), is a human pathogen that causes diverse human diseases including streptococcal toxic shock syndrome (STSS). A GAS outbreak occurred in Brasilia, Brazil, during the second half of the year 2011, causing 26 deaths. Whole genome sequencing was performed using Illumina platform. The sequences were assembled and genes were predicted for comparative analysis with *emm* type 1 strains: MGAS5005 and M1 GAS. Genomics comparison revealed one of the invasive strains that differ from others isolates and from *emm* 1 reference genomes. Also, the new invasive strain showed differences in the content of virulence factors compared to other isolated in the same outbreak. The evolution of contemporary GAS strains is strongly associated with horizontal gene transfer. This is the first genomic study of a *Streptococcal emm 1* outbreak in Brazil, and revealed the rapid bacterial evolution leading to new clones. The emergence of new invasive strains can be a consequence of the injudicious use of antibiotics in Brazil during the past decades.

## Introduction

*Streptococcus pyogenes*, a group A *Streptococcus* (GAS), is a human pathogen. This Gram-positive facultative anaerobe bacterium is responsible for many infections, including pharyngitis, scarlet fever, and cellulitis. GAS is also attributed to life-threatening diseases – such as necrotizing fasciitis as streptococcal toxic shock syndrome (STSS) – and post-infection sequelae, such as Rheumatic fever (Al-ajmi et al., 2012; Hondorp et al., 2012). In past decades, Streptococcal infection, an important health problem, with 660,000 new cases every year (Carapetis et al., 2005). The emergence of more virulent strains, antimicrobial resistance, the increase in the immunologically depleted patient population, and socio-demographic status are factors that facilitate bacterial transmission (Martin et al., 2011; Steer et al., 2012).

Some GAS strains associated with invasive infections can produce exotoxins and specific superantigens that lead to systemic inflammatory responses that result in the most severe infections (Unnikrishnan et al., 2002). Many virulence factors are responsible for the pathogenesis

mechanism, such as M-protein that plays a significant role in phagocytosis evasion (Courtney et al., 2006). The two-component system, known as *covRS*, is responsible for repressing the expression of important virulence factors, such as streptolysin S and streptodornase. Mutations in *covRS* gene may lead to enhanced virulence (Graham et al., 2002).

The whole genome sequencing of different GAS isolates can help to understand the factors that may lead to invasive infections. The samples were collected from patients during an outbreak of invasive *S. pyogenes* that occurred in the city of Brasília, Brazil. Four strains of *S. pyogenes* were isolated from blood of patients with flu-like symptoms, such as high fever, tonsillitis, respiratory failure, and petechiae; and one strain was isolated from the nasal cavity of a patient with pharyngitis. Comparative analysis of the assembled genomes allows the identification of the main virulence factors that could be related to the invasive infection. The data presented here reports the first invasive *S. pyogenes* genomic analysis in South America up to date.

## Materials and Methods

### Outbreak Description and Selected Samples

Bacterial samples were collected during an outbreak of invasive *S. pyogenes* infection that occurred in the city of Brasília, in Brazil, in the period from August to December in 2011, when 101 cases were reported and 26 resulted in deaths. Four samples were isolated from the blood of patients who died due to infection, cultivated in 5% defibrinated sheep blood agar and underwent 24 h incubation 36 ± 1°C with 5% CO_2_. The bacterial species were identified using automated method (MicroScan WalkAway, Siemens Healthcare Systems) according to manufacturer’s instructions. Standard biochemical tests were also used to confirm the identification of bacterial species. Isolates were frozen at -70°C.

The antimicrobial susceptibility testing was also performed according to manufacturer’s instructions (MicroScan WalkAway, Siemens Healthcare Systems) and by the Kirby-Bauer disk diffusion using CLSI procedures. In order to determine the MIC for vancomycin, *E*-test method (bioMerieux Inc., Hazelwood, MO, United States) was used in accordance to the manufacturer’s instructions. The *E*-test MIC readings were performed to conform the dilution scale established by the Clinical and Laboratory Standards Institute for the broth microdilution method. Quality control of susceptibility testing panels from MicroScan was done by testing *Streptococcus pneumoniae* ATCC49619. The antibiotic testing results were interpreted in their susceptibility using the CLSI. *S. pyogenes* strains were typed according to their susceptibility to ampicillin, penicillin, ceftriaxone, cefepime, clindamycin, erythromycin, tetracycline, and vancomycin.

Patients were hospitalized in three different hospitals in Brasília. **Table 1** describes the symptoms manifested by each patient. Patients were anonymized and informed consent was not required. This study was approved by the research ethics committee and registered with the number 16131213.0.0000.5553.

**Table 1:** Description of the symptoms observed in patients infected with invasive and non-invasive *S. pyogenes*.

	Sample name	*emm* type	Sex	Age	Symptoms observed	Treatment given	Time before death
Patient 1	Sp1	1.0	F	32	Flu symptoms, high fever, tonsillitis, respiratory failure, and petechiae on the body.	Metamizole, ceftriaxone, vancomycin	48 h
Patient 2	Sp2	1.0	F	13	Flu symptoms, high fever, tonsillitis, respiratory failure, and petechiae on the body.	Metamizole, ceftriaxone, vancomycin	48 h
Patient 3	Sp3	1.0	F	11	Flu symptoms, high fever, tonsillitis, respiratory failure, and petechiae on the body.	Metamizole, ceftriaxone, vancomycin	Less than 24 h
Patient 4	Sp4	1.0	M	6	Varicella-like rash, fever, bilateral pneumonia, dyspnea, cyanosis, and gastrointestinal bleeding.	Metamizole	Less than 24 h
Patient 5	Sp5	6.4	F	20	Oropharynx infection	Metamizole	


### DNA Extraction

Four selected *S. pyogenes* samples (Sp1–Sp4) had their genomic DNA extracted using a CTAB protocol described previously (Clarke, 2009). The 10 ml of overnight cultures were centrifuged and suspended in 300 μl of CTAB lysis buffer (2% CTAB, 1.4 M NaCl, 100 mM Tris-HCl pH 8.0, 20 mM EDTA and 0.2% mercaptoethanol). The suspensions were left for 30 min in a 65°C dry bath incubator and, after cell lysis, one volume of chloroform: isoamyl alcohol (24:1) was added to the samples. The samples were centrifuged at 10.000 RPM for 10 min and the aqueous phase were transferred to new microtubes. The chromosomal DNAs were precipitated with 0.6 volumes of isopropanol, centrifuged at 10.000 RPM for 5 min, washed with 70% ethanol, and resuspended in 100 μl TE buffer. All procedures were performed in a biosafety level 2 lab. Before DNA sequencing the samples were additionally purified with PowerClean DNA Clean-Up Kit (Mobio).

### Whole Genome Sequencing

The sequencing libraries were constructed using the TruSeq PE Cluster Kit, following the manufacturer instructions. The whole genome of each sample was sequenced with paired-end libraries (2 × 75 bp) to high depth (all above 300×) using TruSeq SBS kit (Illumina) in the Illumina GA II platform at the Federal District High-throughput Genomic Center (Brasília, Brazil).

### Genome Assembly, Alignment, and Phylogeny

Data pre-processing was carried out by removing low-quality reads using the Trimmomatic tool (Bolger et al., 2014). Leading and trailing nucleotides with quality below eight were excluded, as well as ‘N’ bases. A 4-base wide sliding window was set to scan the reads and cut when the average quality per base dropped below 20. All pre-processed reads below 36 bases long were removed.

The high-quality paired-end reads resulted from pre-processing were then used for *de novo* genome assembly of each sample separately using SPAdes (Bankevich et al., 2012). The software Quast (Gurevich et al., 2013) was used to assess the assembly stats and quality.

The assembled genomes were aligned to the *Streptococcus pyogenes MGAS5005* (Accession CP000017.2) and *M1* (Accession AE004092.2) genomes to identify structural variation. The alignment and visualization were performed using the BRIG (Alikhan et al., 2011) tool.

Multiple genome alignments were performed using the DNA sequences of the *S. pyogenes* available in Ensembl Bacteria database and our assembled contigs. The alignment was carried out using Mauve (Darling et al., 2010) and the phylogenetic tree is an output of the progressive alignment performed with the default parameters.

### Nucleotide Sequence Accession Number

The raw sequence data used for the assembly of the genome of each *S. pyogenes* sample (Sp1, Sp2, Sp3, Sp4, and Sp5) have been deposited at NCBI SRA under accession number SRP040580.

### Functional Characterization and Orthologous Groups

The genes were predicted using a locally installed version of GeneMark (Besemer and Borodovsky, 2005). GeneMark suite was used to build the HMM model for prokaryotes using the assembled genomes as references. The produced model was used to generate the GFF file, protein and CDS FASTA files for each genome.

A local orthology clustering was also done using the OrthoMCL software (Li et al., 2003). All predicted proteins from each genome were used as input to perform the analysis, and the procedure was run with default parameters.

The predicted proteins were functionally annotated using a BLAST search in a KEGG Orthology database enriched with UniProt entries (Fernandes et al., 2008).

### MLST Analysis and Virulence Factors Identification

The marker genes for MLST analysis were obtained in the MLST *Streptococcus pyogenes* database^1^ (Jolley and Maiden, 2010). Allele sequences were downloaded and formatted for BLAST (Altschul et al., 1990) search. The assembled contigs were aligned against the alleles database. The MLST was determined by the combination of the best hit with 100% of identity for each marker gene.

A BLAST alignment identified the *emm* gene type among the predicted genes. We used *emm* sequences downloaded from the Centers for Disease Control and Prevention website^2^ as the reference for the search. The best alignment hit with 100% of coverage and identity was used to assign the e*mm* type.

Virulence factors were identified in the predicted proteome using a BLAST search. The reference database was downloaded from VFDB (Chen et al., 2005) and the best alignment for each protein was used as virulence signature.

## Results

Five *S. pyogenes* samples were collected, isolated, and submitted to sequencing. The information about the pathogens, as well as their hosts, is summarized in **Table 1**. The DNA was sequenced, and produced reads covered, in average, 340 times the genome length. The assembled genomes were around 1.8 Mb long. The number of predicted coding genes is around 1800 on the invasive strains. **Table 2** reports the assembly quality measures.

**Table 2:** Assembled genome metrics for each *S. pyogenes* sample.

Sample	Total number of contigs	GC (%)	n50	Length of longest contig	Total bases in contigs	Predicted coding genes	SRA entry
Sp1	25	38.38	118695	715928	1797174	1805	SRR1205846
Sp2	21	38.39	134716	478861	1764153	1746	SRR1238553
Sp3	27	38.38	119191	386637	1804510	1816	SRR1205853
Sp4	22	38.38	119060	636555	1804586	1816	SRR1205858
Sp5	44	38.37	144484	244538	1817645	1930	SRR1205859


**FIGURE 1:**
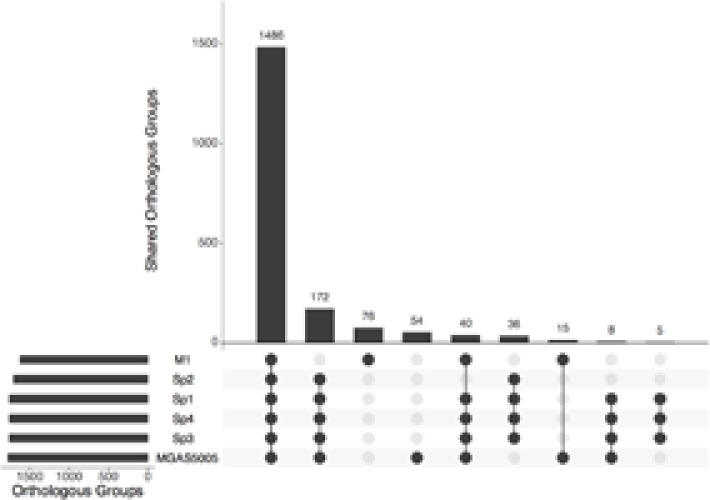
Number of orthologous genes shared six compared strains. Vertical bars show the number of genes shared by the combination of genomes represented in the horizontal axis.

**FIGURE 2:**
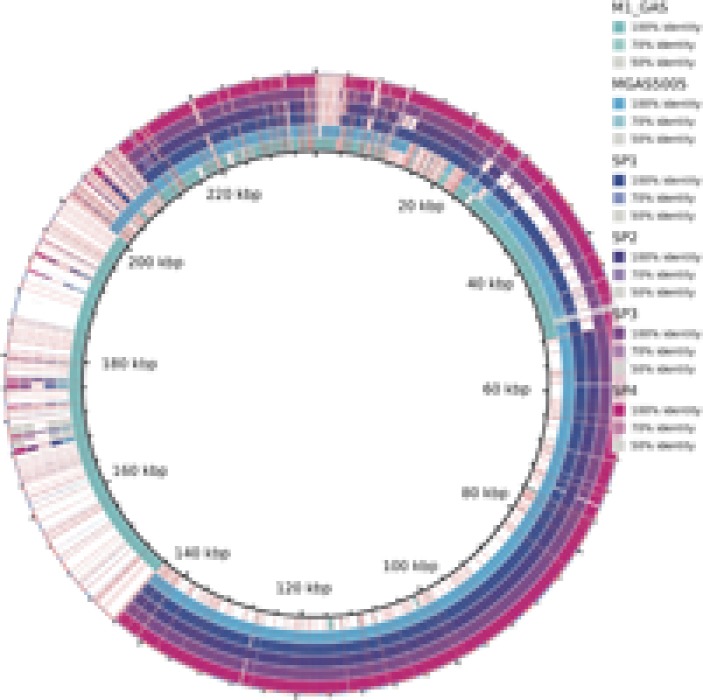
Alignment of non-core genes in Sp1–Sp4, MGAS5005, and M1 GAS strains. Colors represent the alignment identity. The white spaces mean the absence of the gene in the respective genome.

All the predicted proteins from invasive isolates, combined with the proteins from MGAS5005 and M1 GAS, were arranged into 1918 orthologous groups. Clusters comprising proteins from all six isolates summed 1486. One hundred and seventy-two groups contained proteins only from the MGAS5005-like invasive stains. **Figure 1** shows the number of genes present in different genomes. All the non-core genes were aligned to visualize exclusive groups and sequences absent in only one genome. As show in **Figure 2**, there are several exclusive genes from M1 GAS genome, as well as sequences that are missing only in this strain. We could identify 44 genes present exclusively in MGAS5005 genome. Among them, we can identify some genes related to genetic processing: transposase, integrase, relaxase, transcriptional regulator, ribosomal assembly, and energetic metabolism. In isolate Sp2 there were 51 absent genes, most of them are from prophage origin, including the streptodornase *spd3* gene and an antirepressor. The gene products are listed in Supplementary Tables S1 and S2, for MGAS5005 and Sp2 respectively.

The phylogenetic analysis compared the isolated strains with reference *Streptococcus pyogenes* reference genomes from ENSEMBL Bacteria. The whole-genome comparison revealed that Sp1, Sp3, and Sp4 share a common ancestor with the reference strain GA41345 (Supplementary Figure S1). A high similarity can be observed in the cDNA content as well. The Brazilian outbreak isolates Sp1, Sp3, and Sp4 show more similar gene content among each other than when compared to Sp2 (Supplementary Figure S2). The non-invasive strain, Sp5, shares a recent common ancestor with GA19681, an *emm6* strain.

**FIGURE 3:**
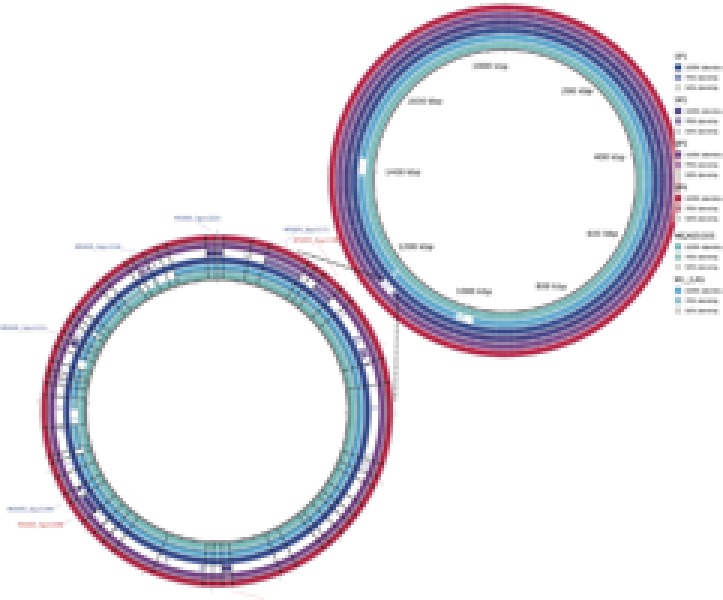
Genomic alignment of invasive strains (Sp1–Sp4) and M1 GAS using MGAS5005 as reference. A 30 kb deletion, at the position 1,200,000 bp, in Sp2 strain corresponds to the Φ5005.2 prophage. Similar deletion can be observed at 1,000,000 bp and 1,400,000 bp positions, in M1 GAS strain, referring to Φ5005.1 and Φ5005.3, respectively. The bottom-left circle expands the Φ5005.2 deletion to highlight the missing genes (white spaces) in Sp2.

The whole genome alignment, using *Streptococcus pyogenes MGAS5005* as the reference, showed that Sp2 is missing a 30 kb region located next to the 1,200,000 bp position of the reference genome (**Figure 3**). This missing fragment has the same genomic location and content as the previously described prophages Φ*5005.2* and Φ*370.3* (Sumby et al., 2005).

**FIGURE 4:**
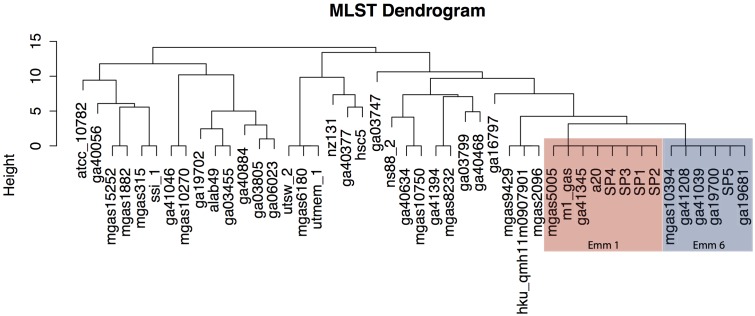
Multilocus sequencing typing (MLST) dendrogram. The invasive outbreak isolates belong to the same sequencing type, which correspond to *emm* type 1 strains.

The multilocus sequencing typing (MLST) approach revealed that Sp1–Sp4, as well as the reference *emm1* strains, belong to the same allele type – Sequence type 28 – for the seven genes used: *gki* 4, *gtr* 3, *muri* 4, *muts* 4, *recp* 4, *xpt* 2, *yqil* 4. The Sp5 strain is the same sequencing type as other five *emm6* strains (**Figure 4**).

**FIGURE 5:**
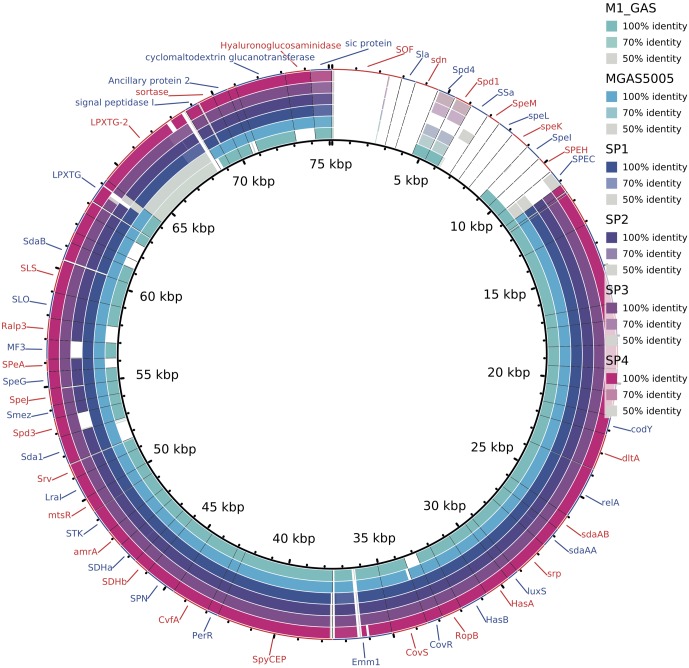
Alignment of predicted genes to virulence factors sequences. Colors, according to the legend, reflect the alignment identity, while white spaces report the absence of that gene. Two genes are missing exclusively in Sp2 genome: *MF3* and *spd3*. LPXTG-2 proteins are conserved exclusively in the outbreak clones, while in M1GAS and MGAS5005 genomes it presents low identity to the reference sequence.

Analyzing the content of virulence factors, we observed that most of the genes are present in all invasive samples (**Figure 5**). The strain Sp2 is slightly different from others due to the absence of two factors: streptodornase and mitogen factor 3 (MF3). Another difference is the presence, only in Brazilian samples, of LPXTG-2 gene that produces an extracellular matrix binding protein.

## Discussion

Group A *Streptococci* infections have been reported as a worldwide public health problem. Several isolates were sequenced and analyzed to better understand the infection mechanism, evolution and to suggest alternatives to slow down the spreading (Nasser et al., 2014). This is the first report of this kind of outbreak in South America.

The information brought from the genome sequencing shows some differences between invasive and non-invasive strains (Nasser et al., 2014). As expected, the pharynx isolate, Sp5, has a different gene content and genomic structure when compared to the invasive isolates. This dissimilarity corroborates with the *emm* type and MLST classification. Moreover, our invasive isolates share a very similar genomic content, with the exception of a 30 kb deletion observed in Sp2. Associated with host factors that are not considered in this study, these genomics structures can be related to the lethality of infection.

The characterization of the outbreak strain, based on comparison with other *emm1* strains, MGAS5005 (Sumby et al., 2005) and M1 GAS (Ferretti et al., 2001), brings some information about the gene content of the clone that generated the Brazilian outbreak. As shown in **Figures 1, 2**, our strains have a similar functional repertoire when compared to the contemporary *emm1* reference. This group, when compared to M1 GAS, acquired two large regions (**Figure 3**). These regions correspond to the prophages Φ5005.1, at position 1,000,000bp, and Φ5005.3, at position 1,400,000 bp (Sumby et al., 2005). Lateral gene transfer is known as a source of innovation in bacteria (Ochman et al., 2000). This event brings ecological adaptation and is related to pathogenesis mechanism. The prophages are linked to the emergence of new invasive strains (Nakagawa et al., 2003). Prophage Φ5005.2 deletion in Sp2 suggests the rise of new GAS clone (**Figure 3**). Pieces of evidence of this episode have recently been reported in six clones among 3,615 sequenced (Nasser et al., 2014), supporting that this is a rare variation. The Φ5005.2 prophage deletion does not strongly change the cellular function since most of the information is phage-related proteins. However, one virulence factor is missing due to this event: streptodornase *spd3*. This gene encodes protein harboring DNAse activity to break the DNA framework of neutrophil extracellular traps (NETs) (Walker et al., 2007; Uchiyama et al., 2012). Another important protein missing is a phage antirepressor that relieves the inhibition promoted by cl-like repressor proteins. It binds to the repressor after initiation of SOS response (Davis et al., 2002). The virulence factor *MF3* is also absent in Sp2 strain (**Figure 5**). The MF3 performs endonuclease activity. Digesting DNA released from dead cells, the enzyme reduces the viscosity of pus and allows the organism greater motility, contributing to the invasion (Bisno et al., 2003). The presence of *SdaB*, another DNase with the same function, balances the absence of *MF3*.

Brazilian isolates contain a protein not identified in previous GAS *emm1* references: LPXTG-2, which is a cell wall anchor domain protein. This extracellular-matrix-binding protein contains eight copies of a conserved domain DUF1542 (pfam07564) associated with antibiotic resistance and cellular adhesion (Clarke et al., 2002). The cited domain is also present in five other *Streptococcus pyogenes* strains: M1, M12, M28, M4, and M49), and in *Streptococcus* and *Staphylococcus* species, that are present in throat infections as well. The cellular adhesion promoted by the LPxTG proteins is essential for a successful infection (Lofling et al., 2011). The LPxTG sequence motif allows covalent linkage of extracellular-matrix proteins with the bacterial peptidoglycan via the activity of sortases (Kreikemeyer, 2004). These proteins are potential targets for drugs and prevention (Flock, 1999).

Although all the *emm1* genomes studied belong to the same MLST group, they have some gene content variation. MGAS5005 has a group of genes that is not present in Brazilian isolates. Our clones miss genes related to genetic information processing and bacterial evolution, such as transposases, relaxase, integrase, ribosomal protein, and chaperone. These genes are not essential to bacterial survival, or are overrepresented in the genome (Jordan et al., 2002). The ribosomal protein L33 is related to 70S ribosome assembly, although its absence does not compromise the ribosomal function (Maguire and Wild, 1997). 4-diphosphocytidyl-2-*C*-methyl-D-erythritol kinase is an essential enzyme for the isoprenoids biosynthesis. In *Mycobacterium tuberculosis*, isoprenoids play a role in the biosynthesis of structural components of the cell wall, being a potential target for antimicrobial drugs (Andreassi et al., 2004; Kang et al., 2011). The usage of antibiotics increase in Brazil during the past decades is 76%, while the world average is 36% (Van Boeckel et al., 2014). This indiscriminate consumption may have led to a strain resistant to these isoprenoids targeted drugs. Other changes are related to energetic metabolism. Tagatose-6-phosphate kinase plays a role in catabolism of lactose and galactose. It also acts as a metabolic sensor, regulating the gene expression by metabolic enzymes, allowing essential transcription programs to be coupled with perceived nutritional status (Loughman and Caparon, 2006). Another missing gene codifies the pyruvate phosphate dikinase. This enzyme is partially deleted in MGAS5005 genome, producing a 69aa product, whereas the protein is 881aa long in *Streptococcus agalactiae 2603*. This full deletion reflects the adaptation in energy metabolism since the bacteria is even more related and adapted to the host.

Regardless of the gain and gene loss events, the isolates maintained their invasive phenotype. This can be explained by the conservation of many important virulence factors with different functions. Some of them are transcription regulators: *Mga*, *Mga2* (Hondorp et al., 2012), *Ralp3* (Kwinn et al., 2007), and *CovR* (Alam et al., 2013). These transcriptional factors are responsible for transcriptional activation of virulence factors such as *Sic*, M protein, *ScpA*, hyaluronic acid capsule, and *Sda* (Kwinn et al., 2007; Hause and McIver, 2012; Alam et al., 2013). The sequenced *covRS* genes had no variations when compared to the reference genomes. In addition, *Ralp3* may also act in epithelial cell invasion and bloodstream survival (Siemens et al., 2012; Alam et al., 2013). In M49 *S. pyogenes* strains, the inactivation of *Ralp3* reduces bacteria attachment and internalization into human keratinocyte. This transcriptional factor controls the expression of several metabolic functions and virulence factors (Siemens et al., 2012). These additional transcriptional factors could provide the invasive strains with higher levels of expression of essential virulence factors in the processes of toxic shock syndrome and, thus, increase their ability to invade other tissues causing invasive infection. In our patients, *S. pyogenes* was isolated directly from blood, indicating a severe invasive infection. The enzyme hyaluronoglucosaminidase catalyzes the breakdown of hyaluronic acid in the host, increasing the permeability in tissues to large molecules (Starr and Engleberg, 2006). *SpeA* is a superantigen that induces the release of pro-inflammatory cytokines by T cells. This high cytokine release is related to the symptoms of *Streptococcal* toxic shock syndrome (Michaelsen et al., 2011). In addition, *SpeA* is able to deflect the host immune response through the activation a range of T-cell subsets, facilitating the establishment of a invasive infection (Maamary et al., 2012). *Sda1* is the main streptodornase of invasive *S. pyogenes* and degrades DNA-based NETs (Walker et al., 2007).

As previously cited, the indiscriminate antibiotics consumption in Brazil increased twice more when compared to worldwide rise. (Van Boeckel et al., 2014). This behavior can explain the rapid evolution and emergence of such different strains in 5 months interval. When compared to MGAS5005, gene loss and prophage deletion can be a consequence of changes to adapt to a specific host and optimize the energy metabolism. In some *Streptococci* strains there is a gene content decay, most of them are not involved in basic cellular processes and tend to fit the metabolism to the available carbon source (Bolotin et al., 2004).

Genomic data reported here have shed some light on the outbreaks caused by *S. pyogenes*. While four strains have virulence factors content highly similar to the MGAS5005 strain, the Sp2 isolate has different genomic organization and virulence factors composition. This suggests the emergence of new *S. pyogenes* clones due to rapid evolution. The MGAS5005 strain is invasive and dispersed worldwide, and this was the first time that a 5005-like outbreak is genetically characterized in South America. Additionally, the great capacity for dispersal of this aggressive bacterium is a serious public health issue. The stress caused by the unselective usage of antimicrobial drugs, which is very common in Brazil, could have led to the rapid evolution and adaptation of this pathogen. The presence of a new LPXTG protein, not observed in MGAS5005 and M1 GAS, is an evidence of a common gain of function for better interaction with the host. This protein can be used as marker to identify these modern 5005-like infections. The genome sequence can be used to develop specific drugs to control the infections. In summary, an improvement in surveillance systems is extremely urgent, so that outbreaks of invasive *S. pyogenes* may be identified and contained in the near future.

## Author Contributions

AB, RA, SA, AV, DG, and GF performed the genomic analysis. FC, MP, FM, and CF-J collected the samples. GF, AB, SD, and OF wrote the manuscript. All authors designed the experiments, read and approved the final manuscript.

## Conflict of Interest Statement

The authors declare that the research was conducted in the absence of any commercial or financial relationships that could be construed as a potential conflict of interest.

## Footnotes

1 http://pubmlst.org/spyogenes/

2 http://www.cdc.gov/streplab/
