# Supplementary material for: Genomic Comparison among Lethal Invasive Strains of Streptococcus pyogenes Serotype M1
Source: Front Microbiol. 2017 Oct 23;8:1993. doi: 10.3389/fmicb.2017.01993 (PMC5660057; doi:10.3389/fmicb.2017.01993)
Supplement: Supplementary file 1 [file Table_1.DOCX]

Supplementary Material

*Streptococcus pyogenes* serotype M1 outbreak in Brazil reveals genomic variations among lethal invasive strains

**Gabriel R. Fernandes, Aulus E. A. D. Barbosa, Renan N. Almeida, Fabíola F. dos S. Castro, Marina de C. P. da Ponte, Celio Faria- Junior, Fernanda M. P. Müller, Antônio A. B. Viana, Dario Grattapaglia, Octavio L. Franco^*^, Sérgio A. Alencar, Simoni C. Dias.**

*** Correspondence:** Corresponding Author: [ocfranco@gmail.com](mailto:ocfranco@gmail.com)

# Supplementary Tables

**Supplementary Table 1:** List of genes observed exclusively in MGAS5005 strain.

| **UniProt Accession** | **UniProt ID** | **Protein names** | **Gene names** | **KO** | **InterPro** |
| --- | --- | --- | --- | --- | --- |
| Q491M5 | Q491M5_STRP1 | 4-diphosphocytidyl-2-C-methyl-D-erythritol kinase (EC 2.7.1.148) | M5005_Spy0074 | K00919; | IPR020568; IPR014721; |
| Q491D4 | Q491D4_STRP1 | Transposase | M5005_Spy0165 | |  |
| Q491B8 | Q491B8_STRP1 | Putative cytosolic protein | M5005_Spy0181 | |  |
| Q491B5 | Q491B5_STRP1 | Putative cytosolic protein | M5005_Spy0184 | |  |
| Q490W5 | Q490W5_STRP1 | Uncharacterized protein | M5005_Spy0234 | |  |
| Q490U0 | Q490U0_STRP1 | Uncharacterized protein | M5005_Spy0259 | |  |
| Q490Q5 | Q490Q5_STRP1 | Transposase | M5005_Spy0294 | |  |
| Q490L6 | Q490L6_STRP1 | Signal peptidase-like protein | M5005_Spy0333 | |  |
| Q490K0 | Q490K0_STRP1 | Uncharacterized protein | M5005_Spy0350 | |  |
| Q490E9 | Q490E9_STRP1 | Putative cytosolic protein | M5005_Spy0401 | |  |
| Q490D8 | RL331_STRP1 | 50S ribosomal protein L33 1 | rpmG1 M5005_Spy0412 | K02913; | IPR001705; IPR011332; |
| Q490A2 | Q490A2_STRP1 | Uncharacterized protein | M5005_Spy0448 | |  |
| Q48ZX0 | Q48ZX0_STRP1 | Putative membrane associated protein | M5005_Spy0481 | |  |
| Q48ZV8 | Q48ZV8_STRP1 | Uncharacterized protein | M5005_Spy0492 | |  |
| Q48ZS7 | Q48ZS7_STRP1 | Uncharacterized protein | M5005_Spy0523 | |  |
| Q48ZF0 | Q48ZF0_STRP1 | Uncharacterized protein | M5005_Spy0650 | |  |
| Q9A060 | Q9A060_STRP1 | Uncharacterized protein | M5005_Spy0721 | | IPR021402; |
| Q48Z46 | Q48Z46_STRP1 | Uncharacterized protein | M5005_Spy0754 | |  |
| Q48YZ9 | Q48YZ9_STRP1 | Relaxase | M5005_Spy0801 | |  |
| Q48YY8 | Q48YY8_STRP1 | Uncharacterized protein | M5005_Spy0812 | |  |
| Q48YR7 | Q48YR7_STRP1 | Transcriptional regulator, LysR family | M5005_Spy0887 | |  |
| Q48YP2 | Q48YP2_STRP1 | Uncharacterized protein | M5005_Spy0912 | |  |
| Q48YI5 | Q48YI5_STRP1 | Uncharacterized protein | M5005_Spy0969 | |  |
| Q48YH6 | Q48YH6_STRP1 | Uncharacterized protein | M5005_Spy0979 | |  |
| Q48YA1 | Q48YA1_STRP1 | Uncharacterized protein | M5005_Spy1053 | |  |
| Q48Y63 | Q48Y63_STRP1 | Transposase | M5005_Spy1091 | | IPR025948; IPR001584; IPR012337; |
| Q48XP4 | Q48XP4_STRP1 | Uncharacterized protein | M5005_Spy1263 | |  |
| Q48XK8 | Q48XK8_STRP1 | Uncharacterized protein | M5005_Spy1299 | |  |
| Q48XI5 | Q48XI5_STRP1 | Uncharacterized protein | M5005_Spy1322 | |  |
| Q48XB1 | Q48XB1_STRP1 | Tagatose-6-phosphate kinase (EC 2.7.1.144) | M5005_Spy1396 | K00917; |  |
| Q48XA4 | Q48XA4_STRP1 | Copper chaperone | M5005_Spy1403 | |  |
| Q48X70 | Q48X70_STRP1 | Uncharacterized protein | M5005_Spy1437 | |  |
| Q48WZ7 | Q48WZ7_STRP1 | Pyruvate, phosphate dikinase (EC 2.7.9.1) | M5005_Spy1510 | K01006; |  |
| Q48WY7 | Q48WY7_STRP1 | Uncharacterized protein | M5005_Spy1520 | |  |
| Q48WW6 | Q48WW6_STRP1 | Uncharacterized protein | M5005_Spy1541 | |  |
| Q48WT3 | Q48WT3_STRP1 | Universal stress protein family | M5005_Spy1574 | |  |
| Q48WR9 | Q48WR9_STRP1 | Uncharacterized protein | M5005_Spy1588 | |  |
| Q48WL5 | Q48WL5_STRP1 | DNA integration/recombination/inversion protein | M5005_Spy1642 | | IPR004107; |
| Q48WK0 | Q48WK0_STRP1 | Putative cytosolic protein | M5005_Spy1657 | |  |
| Q48WJ2 | Q48WJ2_STRP1 | Uncharacterized protein | M5005_Spy1665 | |  |
| Q48WE4 | Q48WE4_STRP1 | Uncharacterized protein | M5005_Spy1713 | |  |
| Q48WD6 | Q48WD6_STRP1 | Uncharacterized protein | M5005_Spy1721 | |  |
| Q48WA5 | RL332_STRP1 | 50S ribosomal protein L33 2 | rpmG2 M5005_Spy1752 | K02913; | IPR001705; IPR011332; |
| Q48W98 | Q48W98_STRP1 | Transcriptional regulator, MutR family | M5005_Spy1759 | |  |

**Supplementary Table 2:** List of genes absent only in Sp2 strain.

| **UniProt Accession** | **UniProt ID** | **Protein names** | **Gene names** | **KO** | **InterPro** |
| --- | --- | --- | --- | --- | --- |
| Q490K4 | Q490K4_STRP1 | Uncharacterized protein | M5005_Spy0346 |  |  |
| Q48XY9 | Q48XY9_STRP1 | Phage protein | M5005_Spy1168 |  |  |
| Q48XY8 | Q48XY8_STRP1 | Streptodornase (EC 3.1.21.1) | spd3 M5005_Spy1169 | K15051; | IPR001604; |
| Q48XY7 | Q48XY7_STRP1 | Putative membrane associated protein | M5005_Spy1170 |  |  |
| Q99Z23 | Q99Z23_STRP1 | Holin (Putative holin-phage associated) | M5005_Spy1172 |  | IPR006485; |
| Q99QD0 | Q99QD0_STRP1 | Phage protein (Uncharacterized protein) | M5005_Spy1173 |  |  |
| Q48XY3 | Q48XY3_STRP1 | Phage protein | M5005_Spy1174 |  | IPR009796; |
| Q48XY2 | Q48XY2_STRP1 | Phage protein | M5005_Spy1175 |  | IPR011675; |
| Q48XY1 | Q48XY1_STRP1 | Phage infection protein | M5005_Spy1176 |  | IPR012892; |
| Q99Z17 | Q99Z17_STRP1 | Phage protein (Uncharacterized protein) | M5005_Spy1177 |  |  |
| Q99Z16 | Q99Z16_STRP1 | Phage protein (Uncharacterized protein) | M5005_Spy1178 |  |  |
| Q99Z15 | Q99Z15_STRP1 | Phage protein (Uncharacterized protein) | M5005_Spy1179 |  |  |
| Q99Z14 | Q99Z14_STRP1 | Phage protein (Uncharacterized protein) | M5005_Spy1180 |  |  |
| Q99Z13 | Q99Z13_STRP1 | Major tail protein (Putative structural protein-phage associated) | M5005_Spy1181 |  |  |
| Q48XX5 | Q48XX5_STRP1 | Phage protein | M5005_Spy1182 |  |  |
| Q99Z11 | Q99Z11_STRP1 | Phage protein (Uncharacterized protein) | M5005_Spy1183 |  |  |
| Q48XX3 | Q48XX3_STRP1 | Phage protein | M5005_Spy1184 |  |  |
| Q99Z09 | Q99Z09_STRP1 | Phage protein (Uncharacterized protein) | M5005_Spy1185 |  | IPR018963; |
| Q99Z08 | Q99Z08_STRP1 | Phage protein (Uncharacterized protein) | M5005_Spy1186 |  | IPR025856; |
| Q99Z07 | Q99Z07_STRP1 | Phage structural protein (Putative structural protein-phage associated) | M5005_Spy1187 |  | IPR024455; |
| Q48XW9 | Q48XW9_STRP1 | Phage protein | M5005_Spy1188 |  | IPR025580; |
| Q99Z05 | Q99Z05_STRP1 | Phage terminase (Uncharacterized protein) | M5005_Spy1189 |  | IPR027417; IPR005021; |
| Q99Z03 | Q99Z03_STRP1 | Phage protein (Uncharacterized protein) | M5005_Spy1191 |  |  |
| Q48XW5 | Q48XW5_STRP1 | Phage protein | M5005_Spy1192 |  |  |
| Q48XW4 | Q48XW4_STRP1 | Phage protein | M5005_Spy1193 |  |  |
| Q99Z00 | Q99Z00_STRP1 | Phage protein (Putative structural protein-phage associated) | M5005_Spy1194 |  | IPR021145; |
| Q99YZ9 | Q99YZ9_STRP1 | Phage protein (Uncharacterized protein) | M5005_Spy1195 |  |  |
| Q99YZ8 | Q99YZ8_STRP1 | HNH endonuclease family protein (Uncharacterized protein) | M5005_Spy1196 |  | IPR002711; IPR003615; |
| Q99YZ7 | Q99YZ7_STRP1 | Phage protein (Uncharacterized protein) | M5005_Spy1197 |  | IPR010861; |
| Q99YZ6 | Q99YZ6_STRP1 | Phage protein (Uncharacterized protein) | M5005_Spy1199 |  |  |
| Q99YZ5 | Q99YZ5_STRP1 | Conserved hypotehetical protein-phage associated (Phage protein) | M5005_Spy1200 |  | IPR009773; IPR012337; |
| Q48XV6 | Q48XV6_STRP1 | Phage protein | M5005_Spy1201 |  |  |
| Q48XV5 | Q48XV5_STRP1 | Phage protein | M5005_Spy1202 |  |  |
| Q48XV4 | Q48XV4_STRP1 | Phage protein | M5005_Spy1203 |  | IPR024432; IPR016974; |
| Q48XV3 | Q48XV3_STRP1 | RecT protein | M5005_Spy1204 | K07455; | IPR018330; IPR004590; |
| Q48XV2 | Q48XV2_STRP1 | Phage protein | M5005_Spy1205 |  |  |
| Q48XV1 | Q48XV1_STRP1 | Phage protein | M5005_Spy1206 |  |  |
| Q48XU9 | Q48XU9_STRP1 | Phage protein | M5005_Spy1208 |  |  |
| Q48XU8 | Q48XU8_STRP1 | DNA replication protein | M5005_Spy1209 |  | IPR006343; |
| Q48XU7 | Q48XU7_STRP1 | Phage replication protein | M5005_Spy1210 |  | IPR011991; |
| Q48XU6 | Q48XU6_STRP1 | Phage protein | M5005_Spy1211 |  |  |
| Q48XU5 | Q48XU5_STRP1 | Excisionase | xis M5005_Spy1212 |  |  |
| Q48XU4 | Q48XU4_STRP1 | Phage protein | M5005_Spy1213 |  | IPR001387; IPR010982; |
| Q48XU3 | Q48XU3_STRP1 | Phage protein | M5005_Spy1214 |  |  |
| Q48XU2 | Q48XU2_STRP1 | Phage protein | M5005_Spy1215 |  | IPR012654; |
| Q48XU0 | Q48XU0_STRP1 | Phage antirepressor protein | M5005_Spy1217 | K07741; | IPR005039; IPR013557; |
| Q48XT9 | Q48XT9_STRP1 | Phage protein | M5005_Spy1218 |  |  |
| Q48XT8 | Q48XT8_STRP1 | Phage transcriptional regulator, Cro/CI family | M5005_Spy1219 |  | IPR001387; IPR010982; |
| Q48XT7 | Q48XT7_STRP1 | Phage protein | M5005_Spy1220 |  | IPR010359; |
| Q48XT6 | Q48XT6_STRP1 | Phage protein | M5005_Spy1221 |  |  |
| Q99YY3 | Q99YY3_STRP1 | Integrase (Putative integrase-phage associated) | int2 int.2 M5005_Spy1222 |  | IPR011010; IPR013762; IPR002104; IPR023109; IPR004107; |

# Supplementary Figures

Supplementary Figure 1. Phylogenetic analysis using genome sequences of *S. pyogenes* strains (Sp1-Sp5) isolated during an invasive infection outbreak occurred in Brasília – Brazil. The other *S. pyogenes* strains were obtained in ENSEMBL Bacteria databank.

Supplementary Figure 2. Phylogenetic analysis using cDNA sequences of *S. pyogenes* strains (Sp1-Sp5) isolated during an invasive infection outbreak occurred in Brasília – Brazil. The other *S. pyogenes* strains were obtained in ENSEMBL Bacteria databank.
